# The feasibility of a national matching service for perfusion education program applicants

**DOI:** 10.1051/ject/2024039

**Published:** 2025-03-07

**Authors:** Blaine Johnson

**Affiliations:** 1 Perfusion Services, UChicago Medicine 5841 S Maryland Ave, Ste E500, MC5040 Chicago IL 60637 USA; 2 Perfusion Education Program, University of Iowa Carver College of Medicine 200 Hawkins Dr, C43-Z GH Iowa City 52242 IA USA

**Keywords:** Perfusion Education Programs, National Matching Services, Quality Improvement, Certification, Accreditation

## Abstract

The perfusion profession is experiencing rapid advancement, creating an array of new opportunities for professional growth and educational expansion. However, this increase in demand is juxtaposed with a concerning limitation in the availability of positions for prospective students and may leave many qualified applicants without admission. This letter explores how implementing a national matching service alongside a centralized application service could streamline the application process for perfusion education programs in the United States. Over the last two decades, the number of available positions in perfusion education programs has surged significantly. This growth presents new challenges in recruitment due to varying requirements and timelines, often resulting in unstable matches. A national matching service could standardize acceptances, mitigate unfair practices, and enhance applicants’ and program decision-making. By ensuring a fair and efficient system, the national matching service could support the growing need for qualified healthcare perfusionists and promote the perfusion profession’s advancement.

## Discussion

The demand for perfusion education programs (PEPs) is increasing rapidly, significantly outpacing the availability of corresponding enrollment positions [1]. This mismatch between the increasing number of applicants seeking admission and the limited openings may result in many qualified candidates without admission. The author has previously discussed the growth of PEPs and the potential benefits of centralized application services (CASs) [2]. This current work further explores how introducing a national matching service (NMS) alongside a CAS could improve the application process for PEPs in the United States, addressing the need for a more efficient and equitable admission process.

The field of perfusion is advancing rapidly, creating new opportunities for growth and expansion in perfusion practice [3]. Recent trends show an increased demand for qualified perfusionists in healthcare, primarily driven by the rise in cardiac surgical procedures and concerns about a potential shortage of perfusionists [4]. The number of positions available for prospective students in PEPs has skyrocketed by 220.6%, rising from 131 in 2001 to 289 in 2024, with an anticipated increase to 311 by 2025 [5, 6].

Navigating the application process can be overwhelming for applicants and admissions committees, primarily due to non-standardized program requirements, differing timelines, and the limited number of available positions. Implementing an NMS could streamline the process of making and accepting appointment offers, saving valuable time for both applicants and programs [7]. In current practice with NMS, the commitment is binding once an applicant is matched, and they must accept the position and start their training.

Since all offers, acceptances, rejections, and final placements occur simultaneously, the NMS provides a fair and standardized way to manage acceptances. An NMS helps eliminate unfair practices common in traditional recruitment processes, such as programs issuing extremely time-sensitive offers or applicants holding onto multiple offers [8]. When an offer is rejected, extending offers to other candidates is often too late, even if alternate choices have been made. The NMS ensures that all decisions are made in a fair and transparent manner, instilling confidence in the integrity of the process [9].

A market characterized by these challenges is unlikely to produce stable matches because there is insufficient time to make mutually beneficial decisions [10]. To expedite the process of making offers, PEPs instituted strict deadlines for responses. These short deadlines, in turn, have compelled students to make early decisions without knowing what other opportunities might arise later.

Numerous platforms employing NMS methodologies are already utilized in healthcare fields such as medicine, pharmacy, dentistry, psychology, and optometry. Traditionally, these platforms have been used to place healthcare residents and interns [11]. However, their services have expanded to include school admissions, student exchanges, and human resources in various sectors, including law and financial services.

Established in 1985, National Matching Services, Inc. pioneered advanced matching software to place physicians into residency positions across the United States [12]. Over the past four decades, the products and services have expanded to encompass all stages of the recruitment process. At the heart of this system is the Roth-Peranson matching algorithm [13]. This algorithm aims to match applicants with their top-ranked programs while also taking into account the programs’ ranking of the applicants, ensuring a mutually satisfying match [14].

One well-known example of an NMS is the entry-level labor market for new physicians in the United States, organized through a centralized clearinghouse [12]. After graduate applicants interview at various residency sites, they create and submit rank order lists (ROLs) that indicate their preferences for the positions they have interviewed for. In parallel, the residency programs submit their ROLs, listing the applicants they interviewed and the number of positions they wish to fill. The NMS then processes these ROLs using an algorithm to match applicants with residency programs [15]. This process is currently completed twice for “The Match” and the “Supplemental Offer and Acceptance Program.”

The use of an NMS has effectively improved a previously chaotic and disorganized process. The Universities and Colleges Admissions Service employs a multiphase matching method in Europe [16]. Based on this model, it was initially suggested that a three-phase system be implemented to address current issues with the Match [17]. This proposed system limits the number of applications that can be submitted initially, establishes a secondary application phase for those who need to apply more broadly, and adds a third phase for the Supplemental Offer and Acceptance Program [18]. The three-phase system could reduce the burden on applicants and programs, ensure a more efficient matching process, and increase the likelihood of stable matches [16].

An integrated CAS and NMS for the perfusion profession would create a centralized platform where applicants could submit a single application to multiple programs. This application could include information and supporting documentation such as official transcripts, personal statements, and letters of recommendation. The clearinghouse would also allow PEPs to transition seamlessly from applications to interview the rankings in a single system [19].

An NMS would streamline and simplify the interview and scheduling process by allowing applicants to self-schedule within a centralized platform. The NMS portal can be tailored to meet each PEP’s specific needs and accommodate various types of interviews, including one-on-one, group, site tours, or multiple rounds of interviews [20]. After interviews, each applicant will submit their confidential ROL, and the matching process will begin.

## Conclusion

The rapid growth of PEPs underscores the urgent need for an efficient and equitable application process for prospective students. Implementing an NMS, in conjunction with CAS, holds significant promise for addressing the disparities between the increasing demand for qualified perfusionists and the limited availability of positions in PEPs. This innovative approach can help mitigate recruitment challenges and pave the way for a more promising future in perfusion education by streamlining the application process and ensuring a fair system for applicants and programs.

## Data Availability

The data supporting this study’s findings are available from the corresponding author upon reasonable request.
